# Modifying the platform-mediated avoidance task: A new protocol to study active avoidance within a social context in rats

**DOI:** 10.1371/journal.pone.0321776

**Published:** 2025-04-29

**Authors:** Cassandra Kramer, Shannon Ruble, Troy D. Fort, Lexe West, Maria M. Diehl

**Affiliations:** Department of Psychological Sciences, Kansas State University, Manhattan, Kansas, United States of America; UFPE: Universidade Federal de Pernambuco, BRAZIL

## Abstract

The platform-mediated active avoidance (PMA) task has been used to study the behavioral and neural mechanisms of a decision-based form of active avoidance. Across 10 days of PMA conditioning, rats learn to avoid a tone-signaled footshock by moving to a safe platform at the cost of forfeiting sucrose reward. Prior studies utilizing the PMA task involved rats learning to avoid alone in a solitary context, but we have recently modified the task to include a social context to determine how social cues may influence PMA acquisition. Additionally, we report novel measures of social interaction that occur during Social Partner PMA conditioning. This protocol provides a detailed methodology for studying active avoidance within solitary and social contexts, as well as key considerations when employing the PMA task.

## Introduction

Avoiding danger is essential for an individual’s survival. Learning that occurs during a dangerous or stressful event can encompass a myriad of contextual and environmental cues that become associated with danger. Previous studies have employed various forms of classical fear conditioning and active avoidance tasks to study the strategies an individual uses to respond to a potential threat [1–3]. Active avoidance occurs when an individual engages in a proactive response to evade a potential threat. Active avoidance may involve the forfeiting of activities associated with survival (such as searching for mates or food) for brief periods of time when it is needed. However, excessive avoidance, especially in the absence of a threat, can become maladaptive as it can interfere with daily activities. Therefore, the appropriate balance of avoidance (avoiding too little and increasing risk versus avoiding too much and missing out on other activities and benefits) is needed, and understanding the behavioral processes which promote or inhibit appropriate avoidance is crucial.

Previous studies have established that the platform-mediated active avoidance (PMA) task is ideal for studying decision-based active avoidance. During the PMA task, rats lever press for a sucrose reward while learning to avoid a tone-signaled footshock by stepping onto a platform. Importantly, the platform is located in a corner opposite from the food area. Therefore, rats must choose to either continue food seeking or actively avoid the impending shock by going to the platform. Thus, this configuration allows us to examine the decision-making process that occurs when weighing pursuit of reward versus safety from danger.

Former research utilizing the PMA task has largely studied avoidance acquisition in a solitary context [4,5] We recently modified the task to study active avoidance within a social context [6], in which we describe the protocol and procedure here. In this variation of the task, the Social Partner PMA task, two rats undergo PMA conditioning simultaneously across 10 days of conditioning. Rats are separated by a perforated, transparent acrylic barrier, which provides access to their own platform, lever, and food dish, while maintaining access to sensory cues from their partner. This design allows us to assess how the presence of social information may influence PMA acquisition while maintaining separate behavioral tracking for each animal. We have previously used the Social Partner PMA task to determine how avoidance and fear-related behaviors differ between social and solitary contexts [6]. Here, we show how these previously measured behaviors change across the course of the tone period on different days of PMA conditioning as well as introduce the idea of an interaction zone in which rats trained in the social context may interact with their partner.

## Materials and methods

The protocol described in this article is included as supporting information (S1 File) and also published on protocols.io (DOI: dx.doi.org/10.17504/protocols.io.rm7vzkq78vx1/v1). Data used to generate Fig 4 is also included as supporting information.

**Fig 1:**
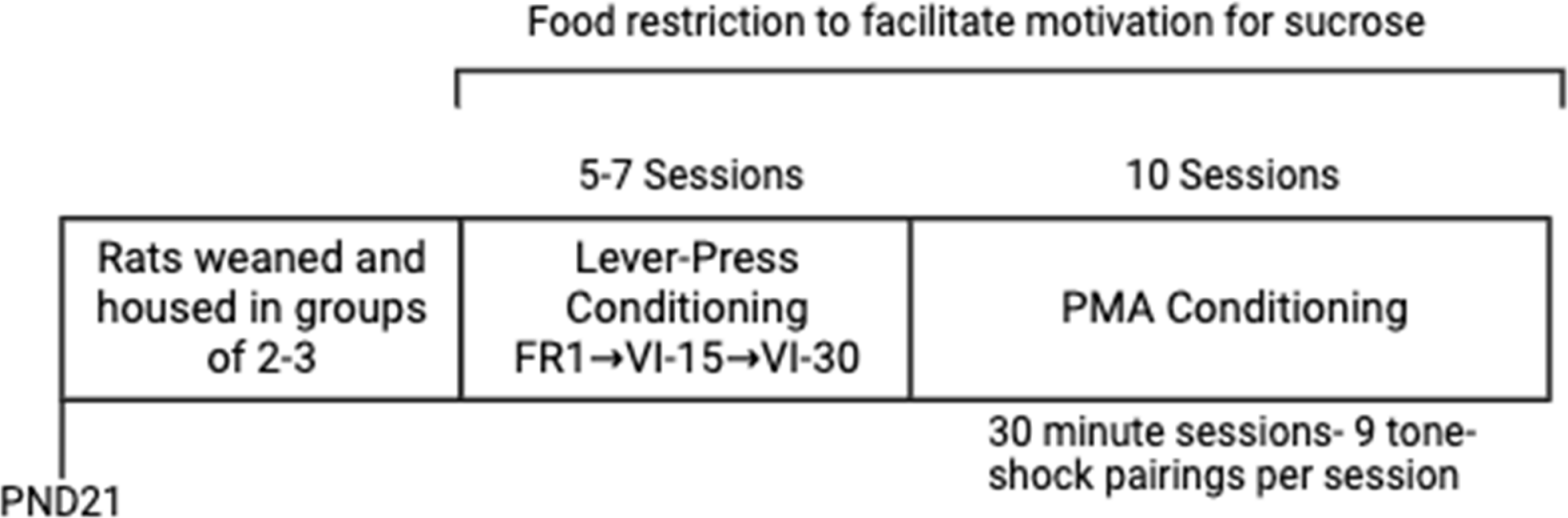
Timeline of Solitary or Social Partner PMA conditioning.

**Fig 2:**
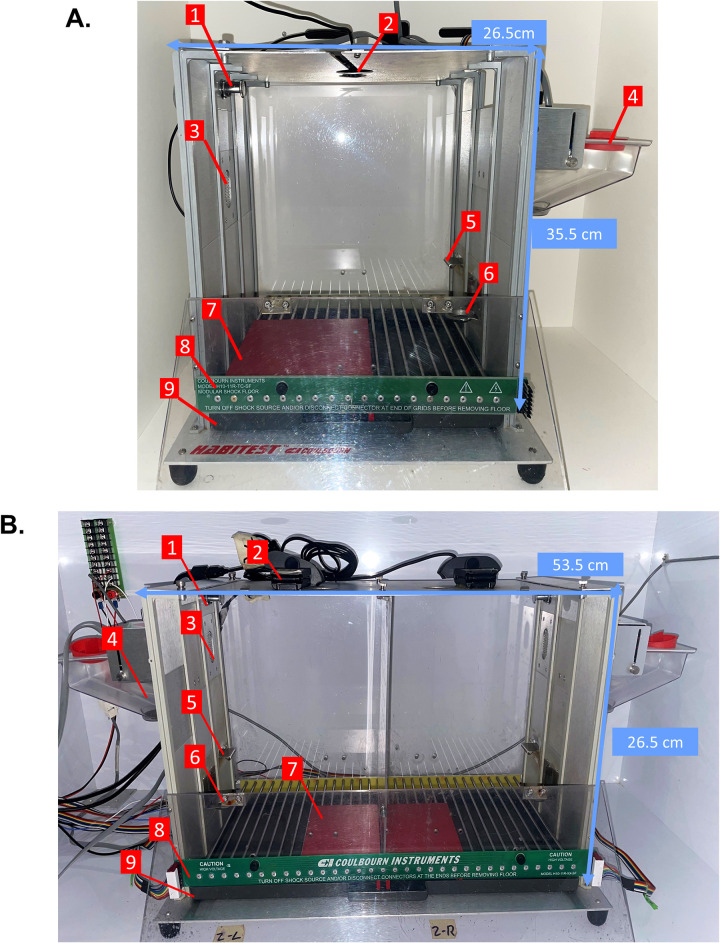
Solitary and Social Partner PMA apparatuses. (A) Conditioning chamber setup for Solitary PMA and (B) Social Partner PMA. The right side of the Social Partner PMA chamber is symmetrical to the left side. (1) LED house light; (2) USB wide-angle camera; (3) Speaker; (4) Pellet dispenser; (5) Lever; (6) Food dish; (7) Platform; (8) Shock floor grid; (9) Drop pan. All measurements are in cm.

**Fig 3:**
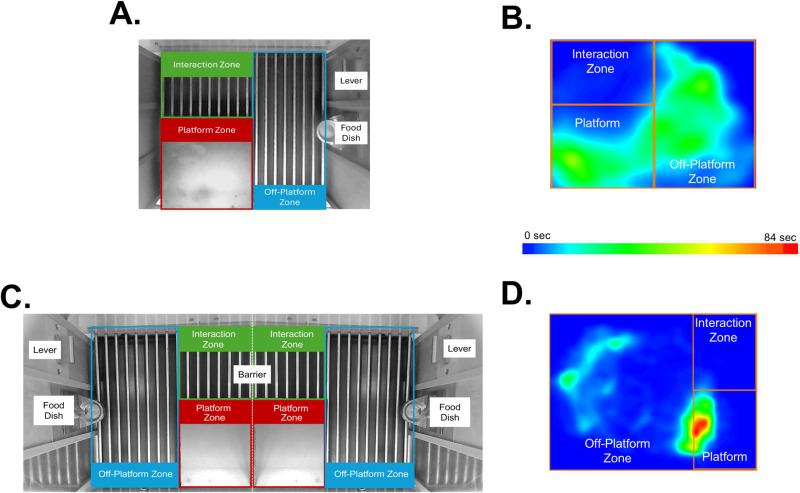
Zone designation and corresponding heat maps in ANY-Maze. (A) Schematic of zones and operant landmarks in Solitary PMA. (B) Example heat map of location averaged across all animals from a single cohort in Solitary PMA conditioning (Male n = 10 and Female n = 6). (C) Schematic of zones and operant landmarks in Social Partner PMA. (D) Example heat map of location averaged across all animals from a single cohort in Social Partner PMA conditioning (Male n = 5 and Female n = 4).

**Fig 4:**
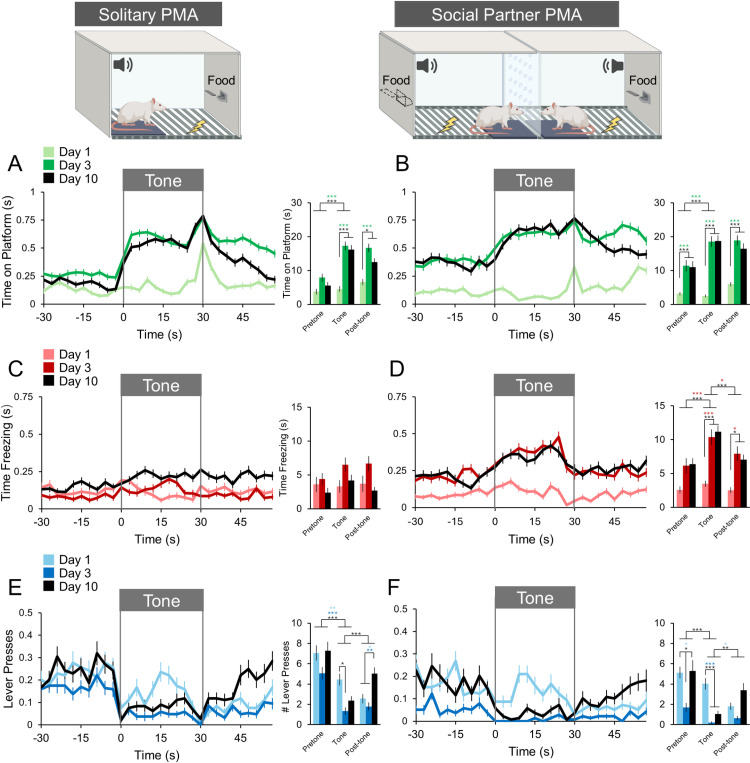
Time-course of behaviors during PMA conditioning. (A-B) Avoidance, (C-D) freezing, (E-F) lever-pressing behaviors at Days 1 (light colored lines), 3 (dark colored lines), and 10 (black lines) of PMA conditioning under Solitary (n=49; 20 females, 28 males) and Social Partner (n=46; 23 females, 23 males) contexts. The first tone period from each session is indicated by the gray box. *p<0.05, **p<0.01, ***p<0.001. See Data for Fig 4 file in Supporting Information for data used to generate this figure.

### Subjects

All procedures were approved by the Institutional Animal Care and Use Committee of Kansas State University in compliance with the National Institutes of Health guidelines for the care and use of laboratory animals. Sprague Dawley rats were bred in-house from rats purchased from a commercial vendor (Charles River Laboratories, Wilmington, MA) and same-sex housed in groups of 2 or 3. Rats were handled and weighed twice a week, or daily if actively participating in an experiment. When all rats within a cage had reached the weight indicated by the standard growth chart for Sprague Dawley rats at approximately 8–10 weeks of age, they were placed on a restricted diet (16–18 g/rat/day) of standard laboratory rat chow to facilitate lever pressing for sucrose pellets while maintaining at least 85% of their target body weight according to the standard growth chart.

### Lever-press conditioning

After being food-restricted, rats were trained to press a lever to receive a sucrose pellet (BioServ, Flemington, NJ) using three shaping schedules (Fig 1). Lever-press conditioning took place over 5–7 days and began at the same time each day. A rat was placed into the same operant box in which it would later undergo either Solitary or Social Partner PMA conditioning (see Fig 2 for box configurations). Further, all rats were run in the same order and within the same operant box to maintain consistent conditions. Each day, prior to the start of the session, boxes were tested to ensure levers and food dispensers were working properly. During the session, the house light remained on while the shock floor grid (shockers off) and platform were present to maintain consistent conditions between lever-press and PMA conditioning. The operant box and surrounding cubicle were closed to minimize outside distractions. Each lever-press conditioning session was 30 minutes.

In phase 1 of lever-press conditioning, rats were placed on a fixed-ratio one (FR-1) reinforcement schedule, receiving one sucrose pellet every time they pressed the lever. Passing criteria for this phase required rats to reach a pressing rate of 2 presses per minute (ppm) by the end of the session. After completion of phase 1, rats were moved to phase 2, where they were placed on a variable-interval reinforcement (VI) schedule receiving a pellet every 15 seconds on average contingent on lever pressing (VI-15). Passing criteria for this phase required rats to reach a pressing rate of 5 ppm by the end of the session. Finally, rats were moved to phase 3, where they were increased to a VI-30 reinforcement schedule, receiving a response-contingent pellet every 30 seconds on average. Passing criteria for this phase required rats to reach a pressing rate of at least 12 ppm by the end of the session. Nearly all rats (98%) that underwent lever-press conditioning (LPC) were successful in learning this task by the seventh day of training. After completing phase 3, rats were ready for Solitary or Social Partner PMA conditioning.

### Solitary PMA procedure

All equipment needed for Solitary PMA is outlined in Table 1 and the configuration is shown in Fig 2A. Multiple pieces of equipment used in this procedure were purchased from Coulbourn Instruments; however, this company no longer produces the equipment. Operant boxes and other materials may be purchased from other vendors or custom built to fit the dimensions specified. Items indicated by an asterisk in Table 1 were purchased from Amazon but could be sourced from elsewhere if made using the same parameters.

**Table 1:** Operant box and equipment needed for Social Partner and Solitary PMA tasks.

Operant Box and Equipment			
Vendor	Item Name	Item Number	Specifications
Coulbourn	Modular Test Cage	H10-11R-TC	This model is specific to rats
Coulbourn	House Light	H11-01R-LED	N/A
Coulbourn	Pellet Feeder	H14-23R	N/A
Coulbourn	Lever	H21-03R	N/A
Coulbourn	Speaker	H12-01R	N/A
Med Associates	Food Dish	ENV-200R1AM	N/A
Coulbourn	Drop Pan	H10-11R-TC-DP	N/A
Coulbourn	Shock Floor	10-11R-TC-SF	N/A
Spedal*	Wide-Angle Webcam	MF920Pro	1080P, 120° viewing angle
**Additional Apparatus and Wiring**			
**Vendor**	**Item Name**	**Item Number**	**Specifications**
Stoelting	ANY-maze Digital Interface	60064	N/A
Stoelting	ANY-maze Relay Interface	60063	N/A
Coulbourn	Programmable Animal Shocker	H21-03R	N/A
Coulbourn	Shock Cable	H93-01–25	Length: 25ft
Evemodel*	Power Distribution Board	PCB005	3 Inputs 2x10 Outputs for DC AC voltage Max voltage: 30V
TUFFIOM*	DC Power Supply	B01NACT99O	Input Voltage: AC 220V/ 110V±10%, 50Hz/ 60HZ Output Voltage: DC 0-30V Output Direct Current: 0-10A
Zulkit*	Electrical junction enclosure box	B07RT6NWTR	Outer Size of electrical enclosure: 7.9“ x 4.7” x 2.95” (L*W*H) Inner Size of electrical enclosure 7.6“x4.4”x2.7” (L*W*H) Thickness: 0.12’‘/3mm
Electronics-Salon*	Power Distribution Fuse Module Board	MD-D1125D-1	Work Voltage: AC or DC 5~32V Total current rating 40Amp Dimensions: 150mm x 72.5mm x 29mm (W x L x H)
MILAPEAK*	18 AWG Gauge Electrical Wire	2468	Color: Red & Black Voltage: 12V DC
Electronix Express*	Hook Up Wire Kit	27WK22STR25	300 volts 22 gauge copper 6 colors 150ft
C&E*	RJ11/RJ12 Keystone jacks	CNE584393	90 degree connection
Meterk	Digital Sound Level Meter	E245122YH	N/A
**Software**			
**Vendor**	**Item Name**	**Item Number**	**Specifications**
Stoelting	ANY-maze video-tracking software	60000	Full-License

*items purchased via Amazon but could be sourced from elsewhere if using the same parameters.

A Rat Modular Test Cage was used as the operant box for the Solitary PMA task (Fig 2A). The operant box included a house light, speaker, lever, and food dish. In the bottom of the operant box, a drop pan and shock floor grid were connected and placed to serve as the floor of the chamber, and to administer foot shocks. A square acrylic platform was placed in the corner opposite the lever and food dish. The legs of the platform extended down through the grid floor to rest on the drop pan underneath, securing it into place over the shock grid. An overhead camera was placed at the top of the operant box for recording the rat during PMA sessions. The operant box was placed inside of a sound-attenuating cubicle to minimize outside noise and visible distractions during the session. The operant chamber and apparatuses were wired into ANY-maze interfaces to allow for communication with the ANY-maze software.

Rats were conditioned with a pure tone (30 sec, 4 kHz, 75 dB) co-terminating with a scrambled footshock delivered through the shock floor grid (2 sec, 0.4 mA). The shortest inter-trial interval (ITI) was approximately 150 seconds while the longest was approximately 200 seconds. All rats received 9 tone-shock pairings per day. Rats were conditioned across 10 days. Access to the lever was unrestricted throughout all conditioning and test sessions, and sucrose reward was given on a VI-30 schedule. The availability of reward on the side opposite to the platform (see Fig 1A) motivated rats to leave the platform during the ITI, facilitating trial-by-trial assessment of avoidance.

Upon the conclusion of each session, the platform, drop pan, and grid were removed and replaced with clean parts after wiping down the inside of the box with deionized water. After all sessions were completed for the day, the box was disinfected with a 70% ethanol solution. Rats were fed following the conclusion of conditioning each day to prevent satiety from affecting lever-pressing during PMA.

### Social Partner PMA procedure

All equipment needed for Social Partner PMA is outlined in Table 1 and the setup is shown in Fig 2B. Any additional equipment needed for Social Partner PMA beyond what is needed for Solitary PMA is listed in Table 2. The operant box for the Social Partner PMA task consisted of a modified Coulbourn shuttle box with the addition of a custom-built transparent, perforated acrylic barrier separating the chamber into two mirrored size-equivalent sides. Each side of the operant box included a house light, speaker, lever, and food dish. In the bottom of the operant box, a drop pan and shock floor grid were connected and placed to serve as the floor of the chamber, and to administer foot shocks. A rectangular acrylic platform was placed in the opposite corner of the lever and food dish, one for each side of the operant box. Two overhead cameras were placed in the top of the operant box, one on each side of the barrier for recording each rat during PMA sessions. The experimental setup is otherwise identical to Solitary PMA.

**Table 2:** Additional operant box and equipment needed only for Social Partner PMA task.

Additional Operant Box and Equipment for Social Partner PMA		
Vendor	Item Name	Item Number
Coulbourn	Operant Shuttle Box	H10-11R-SC
Med Associates	Food Dish	ENV-200R1AM
Coulbourn	Drop Pan	H10-11R-XX-DP
Coulbourn	Shock Floor	10-11R-XX-SF
Med Associates Inc.	Sound-Attenuating Cubicle	ENV-018MD

Rats were assigned a same-sex and age-matched partner. Partner rats were placed one on either side of the perforated acrylic barrier, where they were still able to see, smell, and hear one another. Rats completed 10 days of conditioning within the same operant box and side of barrier with the same partner. Rats within the experiment were food-restricted following the same guidelines as Solitary PMA. Partner rats were conditioned with the same experimental parameters (number of tones, ITI, shock intensity, reinforcement schedule) as described for Solitary PMA. The availability of food on the opposite side of the platform motivated rats to leave the platform during the ITI, facilitating trial-by-trial assessment of avoidance. Cleaning procedures upon the conclusion of conditioning sessions and days were identical to procedures described for Solitary PMA.

### Data collection

ANY-Maze software was used to detect the animal’s location, movements, and behaviors throughout PMA sessions. Sessions were split into pre-tone periods (30s before tone onset), tone periods (30s duration), and shock periods (2s duration, co-terminating with tone periods). All data used in the present protocol are from Ruble et al., 2024 [6].

Several zones were designated to map the rat’s movement throughout the session for both Solitary and Social Partner PMA (Fig 3). A rat was considered to be within a zone when their center of body mass crossed into the specified area. The time that a rat spent within the platform zone during a 30 s tone period was recorded as a measurement of avoidance. The interaction zone was an area adjacent to the platform, next to the perforated barrier, in which rats had the opportunity to interact with or be in close proximity to their partner. An equivalent area that was also adjacent to the platform was delineated in the Solitary PMA task. The number of times a rat pressed the lever was recorded throughout the session via an on/off input: the lever. Rats could lever press freely throughout the session, and the total number of lever presses and rate of presses/minute was recorded at the end of the session.

Freezing behavior was detected across rats by the freezing score algorithm in ANY-maze. If a rat exhibited a freezing score between 50 (freezing “on” threshold) and 70 (freezing “off” threshold), the animal’s behavior was classified as freezing. A minimum freeze duration of 250 ms was required to be calculated as a freezing bout. It is recommended that these parameters be validated with hand scoring and adjusted accordingly.

### Data analysis

Three separate two-way repeated measures ANOVAs were performed to analyze the time spent on the platform, time spent freezing, and the number of lever presses around the tone period. PMA conditioning context (Solitary versus Social Partner) was specified as a between-subjects effect and the time period (pre-tone, tone, post-tone) and day of conditioning (1, 3, 10) were specified as within-subjects effects. Post hoc Tukey tests were used for all pairwise comparisons.

### Troubleshooting

While we do not anticipate any critical issues with the protocol reported above, we have provided solutions to potential issues that experimenters may encounter while running the protocol (see Table 3). For any unanticipated issues, contact the authors for advice.

**Table 3:** Troubleshooting notes for behavioral training during PMA tasks.

Troubleshooting			
Stage	Problem	Possible Reason	Solution
General	A rat is seemingly uninterested in the sucrose reward.	1. The rat is not hungry enough to seek the reward. 2. The rat does not like the sucrose reward.	1. Ensure a rat is not fed prior to behavioral session (LPC or PMA). Adjust homecage food as needed to maintain appropriate levels of motivation for sucrose reward while ensuring IACUC protocols are met. 2. Consider alternate variety of reward (e.g., chocolate-flavored pellet).
General	The rat is moving the platform away from its designated position.	1. The rat is attempting to bring the platform closer to the sucrose reward.	1. The platform can be fixed more rigidly (e.g., duct-taped underneath to the drop pan or grid floor) to further secure it in place.
Lever-Press Conditioning	The rat is not reaching the criterion for passing LPC.	The rat does not understand the association between the lever and receiving a sucrose reward. The rat is not hungry enough to seek the sucrose reward. Rat decreases responding when increased to a leaner schedule of reinforcement (e.g., ratio strain).	1a. Introduce additional shaping procedures to strengthen the relationship between the lever and the reward (ex: crush a sucrose pellet and sprinkle on the lever). 1b. Consider hand-shaping where the experimenter will manually control the pellet delivery via the computer software when the rat gets increasingly closer to pressing the lever. Alternatively, the experimenter can physically guide the rat’s paw to the lever to demonstrate lever pressing for sucrose delivery. 2. Ensure a rat is not fed prior to LPC. Adjust homecage food as needed to maintain appropriate levels of motivation for sucrose reward while ensuring IACUC protocols are met. 3. Rather than continue running the animal on a leaner schedule (e.g., VI-30), revert to VI-15 schedule and run until reliable responding is achieved.
Platform-Mediated Avoidance Conditioning	The rat is not reacting (e.g., not retracting paws, not jumping, not running to platform) to the shock.	The shock level may be too low to elicit a proper behavioral response. The shocker may not be plugged in. Rat may not be making two-points of contact with grid floor.	Raise the shock in increments of 0.02 mA and reassess behavior at each trial. Ensure shocker and grid are assembled properly and working before beginning session. Ensure there is sufficient distance between platform and the lever.
Platform-Mediated Avoidance Conditioning	The rat is not leaving the platform for the majority of the session.	The shock level may be too high to elicit a proper behavioral response. The rat may not be motivated enough to leave the platform.	Lower the shock in increments of 0.02 mA and reassess behavior at each trial. Consider food restricting the rat further, while ensuring IACUC protocols are met.

## Results

Fig 3 shows location data from the defined behavioral zones in the Solitary (Fig 3A) and Social Partner (Fig 3C) contexts. Based on our configuration of these zones between the interaction, platform, and off-platform zones, we extracted heat maps from ANY-Maze showing the allocation of time spent in each zone during PMA conditioning in Solitary (Fig 3B) and Social Partner (Fig 3D) rats. The data presented in the heat maps are averaged across a subset of rats each from a single cohort of animals (Solitary n=16, Social n=9) for one PMA conditioning session.

Qualitatively, rats conditioned under Solitary PMA tended to distribute their time relatively equally across the platform and off-platform zones, while spending little to no time in the Interaction Zone (Fig 3B). However, rats undergoing Social Partner PMA spent markedly more time on the platform near the edge between the Off-Platform and Platform zones and spent some time specifically near the food dish in the Off-Platform Zone. In addition, Social Partner rats tended to show less overall movement when compared to Solitary rats as shown by smaller concentrated areas of time spent within these two zones while still spending little to no time in the Interaction Zone (Fig 3D).

To further characterize behaviors observed during PMA in both solitary and social contexts, we plotted avoidance, freezing, and lever-pressing for the 30 seconds prior to, during, and following the first tone period of PMA during sessions 1, 3, and 10 across Solitary (n=49) and Social Partner (n=46) rats. Fig 4A, B presents avoidance (measured by time spent on the platform) during both contexts of PMA conditioning. Rats conditioned in both contexts increased their avoidance during the tone compared to the pre-tone period on Days 3 (dark green lines) and 10 (black lines) of PMA conditioning (Fig 4A, B, all *p* values <0.001) but not Day 1 (light green lines, *p*=0.999). Social Partner rats spent more time on the platform during the pre-tone period on Days 3 and 10 compared to Day 1 (*p* values <0.001, Fig 4B), a pattern not observed in Solitary rats (Fig 4A). Overall, there were no differences when comparing time spent on the platform between Solitary and Social Partner rats around the first tone period across these PMA conditioning days.

In Fig 4C, D, time spent freezing (regardless of behavioral zone) during both contexts of PMA conditioning are shown. Solitary rats showed less freezing compared to Social Partner rats during the tone period on Day 10 (black lines, *p*<0.001) but not on Day 3 (*p*=0.074) or Day 1 (*p*=1.000). This is in line with Fig 3 which shows that Solitary rats have overall greater locomotion when compared to Social Partner rats. Solitary PMA rats had the same freezing rates during the pre-tone, tone, and post-tone periods across days of PMA conditioning (all *p* values >0.70). In contrast, Social Partner rats increased their freezing during the tone compared to the pre-tone period on Days 3 (dark red) and 10 (black) of PMA conditioning (all *p* values <0.001, Fig 4D), but not Day 1 (light red, *p*=0.910). Freezing decreased from the tone to post-tone period (Day 3, *p*=0.042; Day 10, *p*<0.001), reaching levels similar to those in the pre-tone period.

Finally, Fig 4E, F shows lever pressing during both contexts of PMA conditioning. Lever-pressing was similar between Solitary and Social Partner rats during all time periods on Day 1 (light blue lines, all *p* values >0.800). By Day 3, Solitary rats displayed pre-tone lever-pressing levels similar to those of Day 1 while decreasing their pressing during the tone. Social Partner rats, however, showed significantly lower levels of pre-tone lever-pressing on Day 3 of PMA conditioning compared to Day 1 (*p*=0.011) and maintained this low level of pressing throughout the tone and post-tone periods. It was not until Day 10 of PMA conditioning that Social Partner rats restored their pre-tone lever-pressing levels to that of Day 1 while also decreasing their pressing during the tone (*p*<0.001). On Day 10, both Solitary and Social Partner rats lever-pressed less during the tone compared to the pre-tone period (*p* values<0.001) while resuming lever-pressing at pre-tone levels during the post-tone period.

## Discussion

The PMA task has provided a novel way to study a decision-based form of active avoidance in rodents and has been well-characterized in studies using solitary contexts in both rats [4,5,7–10] and mice [11–17] with minor modifications across labs. For example, some mice studies use nose-poking instead of lever-pressing for reward [15,17] or may instead include a scent exploration component to encourage subjects to leave the platform during the ITI [13,16]. Here, we provide methods for implementing Solitary PMA as it has been previously reported [1,4,9] as well as a new modification of the task that allows rodents to learn simultaneously: Social Partner PMA [6]. By studying rodent behaviors in solitary versus social contexts, we have gained further insight into how social interactions influence behaviors associated with active avoidance.

The results presented here demonstrate that avoidance increases during the tone period over the course of PMA conditioning, regardless of Solitary or Social Partner contexts. The decreased freezing and increased lever-pressing behaviors observed in Solitary rats were further corroborated by the increased locomotion shown in those animals (Fig 3A). This contrasted with the Social Partner rats showing decreased locomotion and increased time in a specific location on the platform (Fig 3B). Despite these differences, time spent on the platform during the tone remained similar across contexts [6]. Here, we also report that rats trained in a social context spent more time freezing exclusively during the tone period – but not before or after – but also more time avoiding outside of the tone period than rats trained in a solitary context (see Fig 4A, B). Rats trained in a social context were also slower to resume lever-pressing after the tone period than rats trained in a solitary context (Fig 4C, D). These findings suggest that a social context may prolong some fear and avoidance behaviors beyond periods in which they are necessary, making them potentially maladaptive. Alternatively, rats trained in a social context may spend longer periods of time on the platform to exchange social cues, given its proximity to the partner rat. Future research using the Social Partner PMA task will provide a greater understanding of how social contexts influence the acquisition and maintenance of avoidance behaviors as well as how social contexts during conditioning may enhance rather than buffer fear.

By implementing Solitary PMA as previous studies have, we can directly compare findings from prior work with this new modification of the task. However, future studies using both Solitary and Social Partner PMA could be carried out exclusively using the Social Partner PMA apparatus. Solitary PMA training could be implemented with only one rat contained within a single side of the Social Partner apparatus across days of training. Doing so could allow for more direct behavioral comparisons between animals trained under social and solitary conditions. While we do not predict that Solitary PMA behaviors would differ if conducted in the Solitary versus Social Partner apparatus, using exclusively the Social Partner apparatus may prove to be a more cost-effective or space-saving solution compared to using both apparatuses.

The Social Partner PMA task can determine how specific social interactions influence learning. Here, we have shown that rats trained in a social context spend more time on the platform than rats trained in a solitary context throughout the entire conditioning session (Fig 3D). Rats trained in a solitary context, however, distribute their time more evenly between the platform and non-platform zones throughout the session (see Fig 3B). This finding could indicate that rats trained in a social context experience conflict between social interaction and food seeking that rats trained in the solitary context do not experience, thus causing Social Partner rats to spend more time near the barrier. We have also introduced the interaction zone as an area where rats trained in Social Partner PMA may interact that does not overlap with the safe platform. While we observed that rats seem to be spending most of their time between the platform and food area in the off-platform zone and very little time in the interaction zone, it is possible that rats trained in the social context are interacting in ways that do not require close proximity to each other. For example, rats may be observing each other from other areas of the chamber or possibly communicating with ultrasonic vocalizations [18–20]. Future studies could measure specific social behaviors such as orienting towards each other, distance between partners, and mimicking certain behaviors that may influence transfer of information and therefore acquisition of Social Partner PMA [21,22].

Additional experimental factors worthy of investigation are further modifications to the location of the platform and the availability of sucrose during Social Partner PMA. As previously discussed, there are two competing motivations in the context of Solitary PMA. Namely, the motivation to obtain sucrose reinforcement directly competes with the motivation to avoid the shock. Importantly, the platform is positioned in such a way that the decision to either obtain sucrose reinforcement or avoid shock is mutually exclusive (i.e., the rat cannot reach the lever while on the platform). However, the context of Social Partner PMA presents an additional competing motivation: the ability to interact with a conspecific in the adjacent operant box. During Social Partner PMA, although avoidance and lever-pressing for sucrose remain mutually exclusive behaviors, social interaction and avoidance via the platform may not, as the rat can interact with the social partner while on the platform. As such, future studies could further parse out how these behaviors are balanced during Social Partner PMA through multiple variations in the task or experimental setup. For instance, to remove the rat’s ability to engage with the conspecific while still avoiding the shock, the platforms could be moved away from each other and in the corner of the chamber opposite of the barrier. This would require the rat to make a mutually exclusive decision regarding whether to avoid the shock at the expense of interacting with their partner or vice versa.

Other future studies could also determine how social behaviors may differ between different types of social partners. Our previous study showed that having a social partner that was previously trained in PMA did not affect PMA acquisition compared to having a social partner that was also naïve at the beginning of PMA conditioning [6]. However, we did not examine the intricate social behaviors mentioned above. It is possible that these different partner types did not affect avoidance behaviors but may have affected the amount of time spent close to the barrier or the amount and/or type of ultrasonic vocalizations emitted. Future studies can use other modifications of partner type such as partners differing by familiarity, social hierarchy, age, sex, strain, etc. to further determine how partner types and specific social interactions may influence PMA acquisition.

Studying Solitary and Social Partner PMA together can provide insight into the processes underlying active avoidance learning alone and in the presence of another individual. Very little research focuses on how individuals learn simultaneously rather than learning from another individual such as in observational fear learning [18,23–26] or fear conditioning by proxy [27–29]. Social Partner PMA is of particular interest because previous research has focused on how the presence of a social partner after an aversive event, such as fear conditioning, alters behavioral responses [30–33]. However, the behavioral effects of a social partner’s presence during an aversive event remain comparatively understudied [6,30]. Tasks such as the Social Partner PMA task described here are, therefore, crucial in understanding how behaviors and neural mechanisms differ between solitary and social contexts, and the parameters outlined can provide consistency as the PMA task evolves.

## Supporting information

S1 File Protocol for the Platform-Mediated Avoidance (PMA) task in rats. (PDF)

S1 Fig Data used to generate Fig 4.
(XLSX)
