# Supplementary material for: Modifying the platform-mediated avoidance task: A new protocol to study active avoidance within a social context in rats
Source: PLoS One. 2025 Apr 29;20(4):e0321776. doi: 10.1371/journal.pone.0321776 (PMC12040205; doi:10.1371/journal.pone.0321776)
Supplement: S1 File — (PDF) [file pone.0321776.s001.pdf]

Mar 15, 2025

Protocol for Social Partner and Solitary Platform-Mediated Avoidance (PMA) Task in Rats

DOI
dx.doi.org/10.17504/protocols.io.rm7vzkq78vx1/v1

Cassandra Kramer[1], Shannon Ruble[1], Troy D. Fort[1], Lexe West[1], Maria Diehl[1]

[1]Kansas State University

Troy D. Fort
Kansas State University

OPEN ACCESS

DOI: dx.doi.org/10.17504/protocols.io.rm7vzkq78vx1/v1

Protocol Citation: Cassandra Kramer, Shannon Ruble, Troy D. Fort, Lexe West, Maria Diehl 2025. Protocol for Social Partner and Solitary Platform-Mediated Avoidance (PMA) Task in Rats. **protocols.io** https://dx.doi.org/10.17504/protocols.io.rm7vzkq78vx1/v1

License: This is an open access protocol distributed under the terms of the **Creative Commons Attribution License**, which permits unrestricted use, distribution, and reproduction in any medium, provided the original author and source are credited

Protocol status: Working
We use this protocol and it's working

Created: January 07, 2025

Last Modified: March 15, 2025

Protocol Integer ID: 119498

Keywords: Platform-Mediated Avoidance (PMA) Task, Rats, Lever-Press Training (LPT) , Operant conditioning, Social , Solitary

Funders Acknowledgements:
NIH
Grant ID: P20-GM103418
NIH
Grant ID: P20-GM113109
NSF
Grant ID: Graduate Research Fellowship (Ruble)

Materials

Biological Materials and Reagents:

- Adult (3-5 months) male and female Sprague-Dawley rats
- Sucrose pellets [1], used for reward
- DI water, for cleaning operant chambers between sessions
- 70% ethanol, for cleaning operant chambers at the end of the day

Operant Box and Equipment – Solitary PMA :

- Coulbourn modular test cage [2] containing a house light [3], pellet feeder [4], lever [5], speaker [6], and food dish [7].
- Drop pan [8] and shock floor grid [9] to place in bottom of operant box
- Square acrylic platforms (custom-built, 14.0 cm each side, 0.33 cm tall)
- Wide-angle webcam [10]
- Custom sound-attenuating cubicle (opaque sides, noise-reducing) for containing operant shuttle box

Note

Coulbourn Instruments is no longer producing equipment. Operant boxes and other materials may be purchased from other vendors such as Med Associates, San Diego Instruments, or can be custom built to fit the dimensions listed above.

Additional Apparatus and Wiring:

- ANY-maze digital interface [11]
- ANY-maze relay interface[12]
- Coulbourn Programmable Animal Shocker [13] and shock cable [14]
- Power Distribution Board [15*]
- DC Power Supply (0-10A/0-30V) [16*]
- Electrical Junction Enclosure Box [17*]
- Panel Mount Power Distribution Fuse Module Board [18*]
- 18 AWG Gauge Electrical Wire [19*]
- Hook Up Wire Kit [20*]
- RJ11/RJ12 Keystone jacks [21*]
- Digital Sound Level Meter for assessing speaker levels [22*]

Software:

- ANY-maze video-tracking software [23]

Biological Materials and Reagents

A	B	C	D	E
**Item**	**Vendor**	**Item Names**	**Item Number**	**Specifications**
1	Bioserv	Dustless Precision Pellets, 50,000/Box	F0021	45 mg, Rodent Purified Diet

Operant Box and Equipment

A	B	C	D	E
**Item**	**Vendor**	**Item Name**	**Item Number**	**Specifications**
2	Coulbourn	Modular Test Cage	H10-11R-TC	This model is specific to rats
3	Coulbourn	House Light	H11-01R-LED	N/A
4	Coulbourn	Pellet Feeder	H14-23R	N/A
5	Coulbourn	Lever	H21-03R	N/A
6	Coulbourn	Speaker	H12-01R	N/A
7	Med Associates	Food Dish	ENV-200R1AM	N/A
8	Coulbourn	Drop Pan	H10-11R-TC-DP	N/A
9	Coulbourn	Shock Floor	10-11R-TC-SF	N/A
10	Spedal*	Wide-Angle Webcam	MF920Pro	1080P, 120° viewing angle

Additional Apparatus and Wiring

A	B	C	D	E
**Item**	**Vendor**	**Item Name**	**Item Number**	**Specifications**
11	Stoelting	ANY-maze Digital Interface	60064	N/A
12	Stoelting	ANY-maze Relay Interface	60063	N/A
13	Coulbourn	Programmable Animal Shocker	H21-03R	N/A
14	Coulbourn	Shock Cable	H93-01-25	Length: 25ft
15	Evemodel*	Power Distribution Board	PCB005	• 3 Inputs<br>• 2x10 Outputs for DC AC voltage<br>• Max voltage: 30V
16	TUFFIOM*	DC Power Supply	B01NACT99O	• Input Voltage: AC 220V/ 110V±10%, 50Hz/ 60HZ<br>• Output Voltage: DC 0-30V<br>• Output Direct Current: 0-10A
17	Zulkit*	Electrical junction enclosure box	B07RT6NWTR	• Outer Size of electrical enclosure: 7.9 x 4.7 x 2.95 (L*W*H)<br>• Inner Size of electrical enclosure 7.6x4.4x2.7 (L*W*H)<br>• Thickness: 0.12"/3mm

A	B	C	D	E
18	Electronics -Salon*	Power Distribution Fuse Module Board	MD-D1125D-1	• Work Voltage: AC or DC 5~32V<br>• Total current rating 40Amp<br>• Dimensions: 150mm x 72.5mm x 29mm (W x L x H)
19	MILAPEAK *	18 AWG Gauge Electrical Wire	2468	• Color: Red & Black<br>• Voltage: 12V DC
20	Electronix Express*	Hook Up Wire Kit	27WK22STR25	• Authentic Stranded •<br>300 volts • 22<br>gague copper<br>• 6 colors •<br>150ft
21	C&E*	RJ11/RJ12 Keystone jacks	CNE584393	• 90 degree connection
22	Meterk	Digital Sound Level Meter	E245122YH	N/A

Software

A	B	C	D	E
**Item**	**Vendor**	**Item Name**	**Item Number**	**Specifications**
23	Stoelting	ANY-maze videotracking software	60000	Full-License

*Items indicated by an asterisk were purchased from Amazon, but could be sourced from elsewhere if items are made using the same parameters

Operant Box and Equipment – Social Partner PMA

- Coulbourn operant shuttle box [24] containing two house lights [3], two pellet feeders [4], two levers [5], two speakers [6], and two food dishes [7], one on each side of the shuttle box.
- Plexi-glass, perforated divider for separating sides of operant box (custom-built, 28.24 cm x 25.17 cm x 0.32 cm, 0.48 cm holes)
- Drop pan [25] and shock floor grid [26] to place in bottom of operant box
- Two rectangular acrylic platforms (custom-built, 10x15 cm), one per side of the operant box
- Two wide-angle webcams [10*], one per side of the operant box
- Sound-attenuating cubicle [27] (noise-reducing) for containing operant box

Additional Apparatus and Wiring:

- ANY-maze digital interface [11]
- ANY-maze relay interface[12]
- Coulbourn Programmable Animal Shocker [13] and shock cable [14]
- Power Distribution Board [15*]
- DC Power Supply (0-10A/0-30V) [16*]
- Electrical Junction Enclosure Box [17*]

- Panel Mount Power Distribution Fuse Module Board [18*]
- 18 AWG Gauge Electrical Wire [19*]
- Hook Up Wire Kit [20*]
- RJ11/RJ12 Keystone jacks [21*]
- Wire Crimping Tool
- Digital Sound Level Meter for assessing speaker levels [22*]

Software

ANY-maze video-tracking software [23]

Operant Box and Equipment Unique to Social Partner PMA

A	B	C	D	E
Item	Vendor	Item Name	Item Number	Specifications
24	Coulbourn	Operant Shuttle Box	H10-11R-SC	This model is specific to rats
25	Coulbourn	Drop Pan	H10-11R-XX-DP	N/A
26	Coulbourn	Shock Floor	10-11R-XX-SF	N/A
27	Med Associates Inc.	Sound-Attenuating Cubicle	ENV-018MD	N/A

Note

Biological materials and reagents, additional apparatus and wiring, and software are identical to those specified in Table 1
